# Segmentectomy and Wedge Resection for Elderly Patients with Stage I Non-Small Cell Lung Cancer: A Systematic Review and Meta-Analysis

**DOI:** 10.3390/jcm11020294

**Published:** 2022-01-06

**Authors:** Peiyu Wang, Shaodong Wang, Zheng Liu, Xizhao Sui, Xun Wang, Xiao Li, Mantang Qiu, Fan Yang

**Affiliations:** Department of Thoracic Surgery, Peking University People’s Hospital, Beijing 100044, China; 18339979852@163.com (P.W.); 13651217347@163.com (S.W.); pkuliu1123@163.com (Z.L.); suixizhao@pkuph.edu.cn (X.S.); xungeshiwo@163.com (X.W.); dr.lixiao@163.com (X.L.); qiumantang@163.com (M.Q.)

**Keywords:** non-small cell lung cancer, lobectomy, segmentectomy, wedge resection, elderly patients

## Abstract

Background: Considerable controversies exist regarding the efficacies of segmentectomy and wedge resection for elderly patients with early-stage non-small cell lung cancer (NSCLC). This systematic review and meta-analysis aimed to solve these issues. Methods: We searched the online databases PubMed, Web of Science, EMBASE, and Cochrane Library to identify eligible studies. Elderly patients were defined as ≥65 years. Early-stage NSCLC was defined as stage I based on TNM systems. The primary endpoints were survival outcomes (overall survival (OS), cancer-specific survival (CSS), and disease-free survival (DFS)) and recurrence patterns. The second endpoints were perioperative morbidities. The hazard rate (HR) and odds ratio (OR) were effect sizes. Results: Sixteen cohort studies (3140 participants) and four database studies were finally included. Segmentectomy and lobectomy showed no significant difference in OS (cohort studies HR 1.00, *p* = 0.98; database studies HR 1.07, *p* = 0.14), CSS (HR 0.91, *p* = 0.85), or DFS (HR 1.04, *p* = 0.78) in elderly patients with stage I NSCLC. In contrast, wedge resection showed inferior OS (HR 1.28, *p* < 0.001), CSS (HR 1.17, *p* = 0.001) and DFS (HR 1.44, *p* = 0.042) compared to lobectomy. Segmentectomy also showed comparable local recurrence risk with lobectomy (OR 0.98, *p* = 0.98), while wedge resection showed increased risk (OR 5.46, *p* < 0.001). Furthermore, sublobar resections showed a decreased risk of 30/90-day mortality, pneumonia, and leak complications compared to lobectomy. Conclusion: Segmentectomy is promising when applied to elderly patients with stage I NSCLC, while wedge resection should be limited. Randomized controlled trials are warranted to validate these findings.

## 1. Introduction

As the second most common cancer and the leading cause of cancer-related mortality, lung cancer is threatening the livelihoods and health of the global population [1]. With the generalization of screening strategies for risk populations, an increasing number of lung cancers are detected in the early stage. Anatomic lobectomy with hilar and mediastinal lymph node dissection has been listed as the standard treatment for clinical stage I non-small-cell lung cancers (NSCLCs) [2]. Sublobar resections, including segmentectomy and wedge resection, are currently performed for early-stage peripheral tumors or cases of impaired cardiopulmonary function [3,4]. The merits of sublobar resections are mainly reducing perioperative morbidities and preserving postoperative pulmonary function [5,6].

Increased evidence supports the sublobar resections as alternatives to lobectomy for early-stage NSCLC. Ijsseldijk et al.’s meta-analysis demonstrated equivalent overall survival (OS) of sublobar resections with lobectomy for stage IA1 NSCLC [7]. Segmentectomy, with better parenchymal margin and lymph node staging than wedge resection, achieved equivalent OS, cancer-specific survival (CSS), and disease-free survival (DFS) with lobectomy for stage IA1-2 NSCLC [8]. Segmentectomy was also reported to achieve comparable survival and recurrence patterns with lobectomy for adult patients with stage IA3 but not stage IB NSCLC [9,10].

Considering the increased comorbidities and degenerated cardiopulmonary function during the aging process, sublobar resections are anticipated to benefit elderly patients with stage I NSCLC. However, considerable controversies exist regarding the efficacies of sublobar resections for this population. Although the analyses of the STS General Thoracic Surgery Database (GTSD) [11] and the Surveillance, Epidemiology, and End Results (SEER) database 3 demonstrated inferior efficacy of sublobar resections, several cohort studies [12,13,14] reported satisfactory outcomes. In particular, conceivable differences in therapeutic efficacy exist between segmentectomy and wedge resection for elderly patients [15,16]. Before the disclosure of a randomized controlled study (STEPS) [17], a systematic review and meta-analysis is anticipated to help solve these issues.

This systematic review and meta-analysis investigated the efficacies of segmentectomy and wedge resection for elderly patients with stage I NSCLC with lobectomy as a comparator. The survival outcomes and recurrence patterns were primarily studied.

## 2. Materials and Methods

This systematic review and meta-analysis was registered with the PROSPERO International Prospective Register for Systemic Reviews (CRD42021246333) and performed according to the Preferred Reporting Items for Systematic Review and Meta-Analysis (PRISMA) guidelines.

### 2.1. Data Sources and Searches

Two reviewers (P.W. and Z.L.) independently conducted a systematic and comprehensive literature search of online databases PubMed, Web of Science, EMBASE, and Cochrane Library (title and abstract) to identify cohort studies and database studies performed before 1 April 2021 that simultaneously compared the efficacies of sublobar resections (segmentectomy and wedge resection) with lobectomy for elderly patients with stage I NSCLC. The search strategy combined search terms of lung cancer, lung adenocarcinoma, lung squamous cell carcinoma, lobectomy, segmentectomy, wedge resection, sublobar resection, survival, recurrence, mortality, and complications (the search strategy for PubMed is shown in Supplemental Table S1). After 1 April 2021, the literature update was performed manually on a weekly basis until 10 November 2021. The reference lists of all included articles were checked to identify other relevant articles. Any disagreement was resolved by the advisory group consisting of three senior authors (S.W., X.L. and F.Y.).

### 2.2. Study Selection

The inclusion criteria were as follows: (1) study population: elderly NSCLC patients (≥65 years) with stage I NSCLC (the eighth version of the TNM system was preferred to the seventh and sixth versions of the TNM system); (2) comparison: segmentectomy, wedge resection, or sublobar resection vs. lobectomy; (3) endpoints: perioperative morbidities, recurrence patterns, or survival outcomes; and (4) study type: prospective or retrospective cohort studies and database studies. The exclusion criteria included: (1) the lack of independent data of elderly patients; (2) the lack of comparison between sublobar resections and lobectomy; (3) the inclusion of advanced NSCLC; (4) reviews, case reports, comments, editorials, or corresponding letters; (5) overlapping studies; and (6) non-English literature. Two authors (P.W. and Z.L.) independently reviewed the titles and abstracts to screen possibly eligible articles. The full text was then independently reviewed for final validation. During these processes, any disagreement was resolved by the adversary group.

### 2.3. Data Extraction

The Cochrane Good Practice data extraction template was used to establish a standardized form for data extraction. Data on the study design, study period, sample, patient characteristics, disease characteristics, surgical approaches, operative parameters, perioperative morbidities (complications, mortality), survival outcomes (OS, CCS, RFS), and recurrence patterns (local recurrence, distant metastasis) were independently extracted by two authors. Any discrepancy was resolved by checking the original articles. 

### 2.4. Quality Assessment

Quality assessments were performed using the Newcastle Ottawa Scale (NOS). The NOS includes six aspects, eight scoring points, and a total score of 9 points. A study with a total score of ≥7 points was regarded as high quality [18]. Furthermore, the quality of the quantitatively pooled outcomes was determined with the Grading of Recommendation Assessment, Development, and Evaluation (GRADE) system [19]. The GRADE system includes five negative domains (risk of bias, inconsistency, indirectness, imprecision, and publication bias) and allocates the pooled outcomes with scores ranging from 1 (very low quality) to 4 (high quality).

### 2.5. Endpoints

The primary endpoints were survival efficacy (OS, CSS, and DFS) and recurrence patterns of segmentectomy and wedge resection compared to lobectomy. The secondary endpoints were perioperative mortality and complications.

### 2.6. Statistical Analysis

The summary statistics included odds ratios (ORs) with 95% confidence intervals (CIs) for categorical data, weighted mean differences (WMDs) with 95% CIs for continuous data, and hazard rates (HRs) with 95% CIs for survival data. The estimated survival data were extracted using Parmar et al.’s and Williamson et al.’s methods [20,21]. The between-study heterogeneity was estimated with Cochran’s Q statistic using chi-square and *I*^2^ statistics. The fixed-effects model was used for low to moderate heterogeneity (*I*^2^ ≤ 50%), while the random-effects model was used for high heterogeneity (*I*^2^ > 50%). The quantitative analyses of database studies and cohort studies were generally separated to avoid data overlapping. Funnel plots and Egger’s test were used to assess publication bias. Sensitivity analysis was conducted by omitting one study at a time. A two-tailed *p* value < 0.05 was considered statistically significant. All analyses were conducted with STATA version 12 software (Stata Corporation, College Station, TX, USA). 

## 3. Results 

### 3.1. Study Selection and Quality Assessment

**Figure 1:**
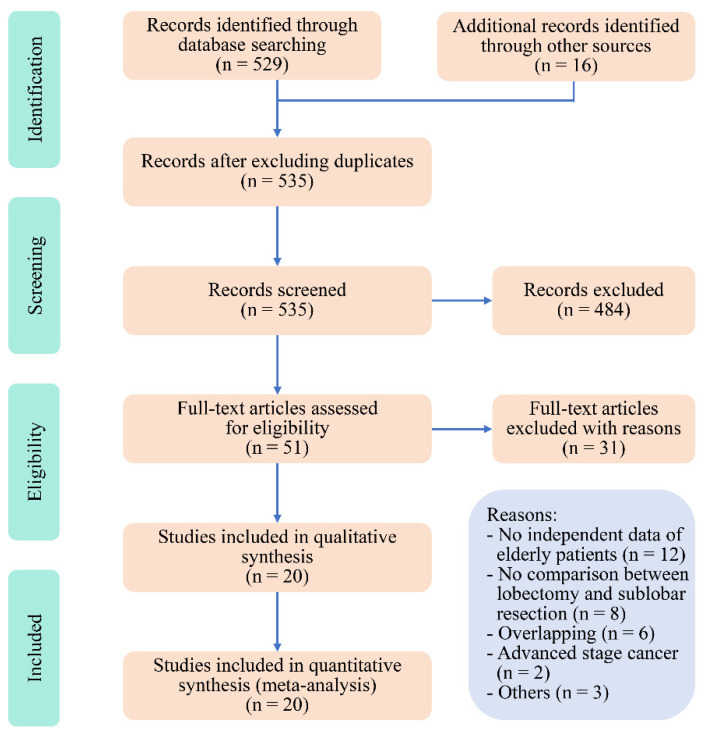
Preferred Reporting Items for Systematic Review and Meta-Analysis (PRISMA) flow diagram for study selection.

After searching the mentioned online databases and inspecting reference lists, the primary literature review identified 535 papers (Figure 1). The review of title and abstract excluded 484 papers. After evaluating the full texts, 4 database studies [3,4,5], 11 and 16 cohort studies [12,13,14,22,23,24,25,26,27,28,29,30,31,32,33,34] were included. The cohort studies included 3140 participants with 2009 lobectomies and 1131 sublobar resections. The list of excluded studies with reasons is shown in Supplemental Table S2. The NOS demonstrated a high quality of 10 studies and moderate quality of the remaining studies. (Supplemental Table S3).

### 3.2. Study Characteristics

The database studies reported the survival or mortality outcomes of elderly patients with stage I NSCLC from the SEER, STS-GTSD, or National Cancer Database (NCDB) (Table 1). The cohort studies were all retrospectively conducted with four studies using propensity score matching. Ten studies included early-stage NSCLC based on pathological cancer stage, while six studies included patients based on clinical cancer stage. The definition of elderly patients varied across these studies: 80 years (four studies), 75 years (four studies), 70 years (five studies), and 65 years (three studies). Sublobar resections were mostly adopted for patients with impaired cardiopulmonary function or intentionally introduced for early-stage peripheral tumors. Systematic lymph node dissection or sampling was commonly implemented during lobectomy and segmentectomy but not during wedge resection. 

Regarding patient characteristics, the sublobar resection groups showed impaired pulmonary function and increased chronic obstructive pulmonary diseases compared to the lobectomy group (Table 1). The database analysis of SEER demonstrated more advanced age in sublobar resection groups [3], but this was not commonly reported in cohort studies. Regarding the disease characteristics, the tumor was larger in the lobectomy group than in the sublobar groups, with significant differences being reported in six studies [3,4,14,23,31,33]. The sublobar resection groups were also reported to have less lymph node dissection [12,24,31].

### 3.3. Primary Endpoints: Overall Survival (OS), Cancer-Specific Survival (CSS), and Disease-Free Survival (DFS)

**Figure 2:**
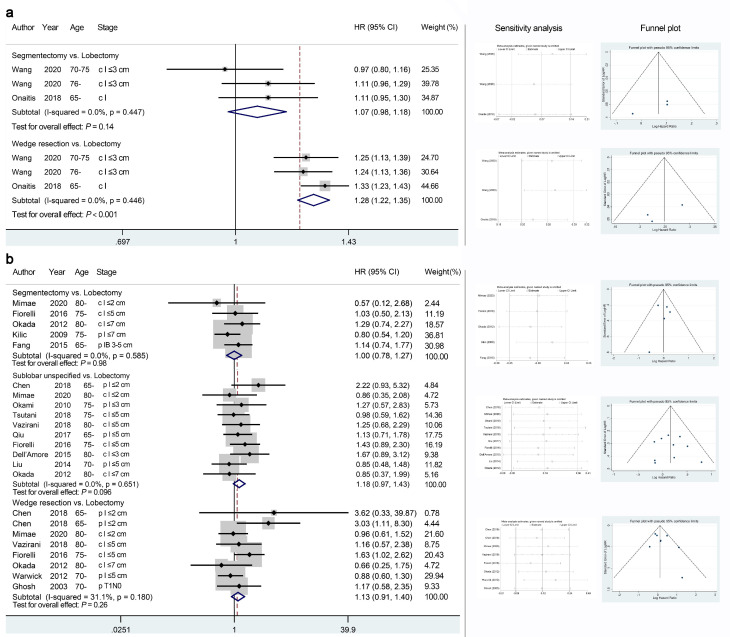
Meta-analyses comparing the overall survival outcomes of sublobar resections with those of lobectomy using database studies (**a**) or cohort studies (**b**). The right panel presents the outcomes of sensitivity analyses and the funnel plots corresponding to the forest plots shown in the left panel. HR, hazard ratio; CI, confidence interval.

The quality of survival-related quantitative analyses was mostly moderate according to the GRADE system (Table 2). Two database studies and 14 cohort studies analyzed the effects of sublobar resections on OS, CSS, or DFS for elderly patients with stage I NSCLC. The quantitative analyses of database studies demonstrated non-significantly poorer OS in the segmentectomy group (Figure 2a, HR 1.07, 95% CI 0.98–1.18, *p* = 0.14; *I*^2^ = 0; Egger’s test, *p* = 0.19) and significantly poorer OS in the wedge resection group (HR 1.28, 95% CI 1.22–1.35, *p* < 0.001; *I*^2^ = 0; Egger’s test, *p* = 0.30) than in the lobectomy group. In contrast, the pooled analyses of cohort studies demonstrated no significant difference in OS between segmentectomy and lobectomy (Figure 2b, HR 1.00, 95% CI 0.78–1.27, *p* = 0.98; *I*^2^ = 0; Egger’s test, *p* = 0.84) but non-significantly poorer OS of wedge resection than lobectomy (HR 1.13, 95% CI 0.91–1.40, *p* = 0.26; *I*^2^ = 31.1; Egger’s test, *p* = 0.29). The unspecified sublobar resection group also showed non-significantly poorer OS than the lobectomy group (HR 1.18, 95% CI 0.97–1.43, *p* = 0.096; *I*^2^ = 0; Egger’s test, *p* = 0.81). 

**Figure 3:**
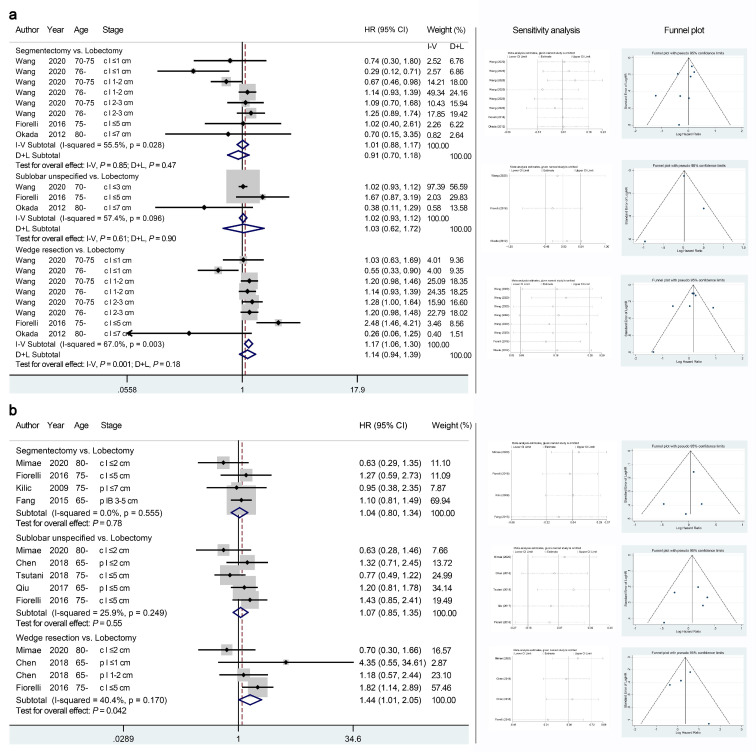
Meta-analyses comparing the cancer-specific survival (**a**) and disease-free survival (**b**) outcomes of sublobar resections with those of lobectomy. The right panel presents the outcomes of sensitivity analyses and the funnel plots corresponding to the forest plots shown in the left panel. HR, hazard ratio; CI, confidence interval. I-V: fixed-effects model; D+L: random-effects model.

No significant difference in CSS was observed between segmentectomy and lobectomy for elderly patients with stage I NSCLC (Figure 3a, random-effect, HR 0.91, 95% CI 0.70–1.18, *p* = 0.85; *I*^2^ = 55.5; Egger’s test, *p* = 0.15). Although the meta-analysis using a fixed-effects model demonstrated significantly poorer CSS in the wedge resection group than in the lobectomy group (HR 1.17, 95% CI 1.06–1.30, *p* = 0.001; *I*^2^ = 67.0; Egger’s test, *p* = 0.44), the difference was not significant when using a random-effects model. In particular, the multidimensional analyses of the SEER database demonstrated better CSS of segmentectomy and wedge resection than lobectomy for IA1 NSCLC patients over 75 years (HR 0.29, 95% CI 0.12–0.71; HR 0.55, 95% CI 0.33–0.90, respectively) [3].

Regarding DFS (Figure 3b), the meta-analysis demonstrated no significant difference between segmentectomy and lobectomy (HR 1.04, 95% CI 0.80–1.34, *p* = 0.78; *I*^2^ = 0; Egger’s test, *p* = 0.56), while wedge resection showed a significantly poorer prognosis (HR 1.44, 95% CI 1.01–2.05, *p* = 0.042; *I*^2^ = 40.4; Egger’s test, *p* = 0.92). Segmentectomy, unspecified sublobar resection, and wedge resection (vs. lobectomy) showed gradually exacerbated DFS. 

Regarding the direct comparison between segmentectomy and wedge resection (Supplemental Figure S1), segmentectomy showed better OS (HR 0.80, 95% CI 0.71–0.90, *p* < 0.001) and CSS (HR 0.77, 95% CI 0.65–0.91, *p* < 0.001) but not DFS (HR 0.98, 95% CI 0.56–1.71, *p* = 0.95). No significant heterogeneity or publication bias was detected.

### 3.4. Primary Endpoints: Recurrence Patterns 

**Figure 4:**
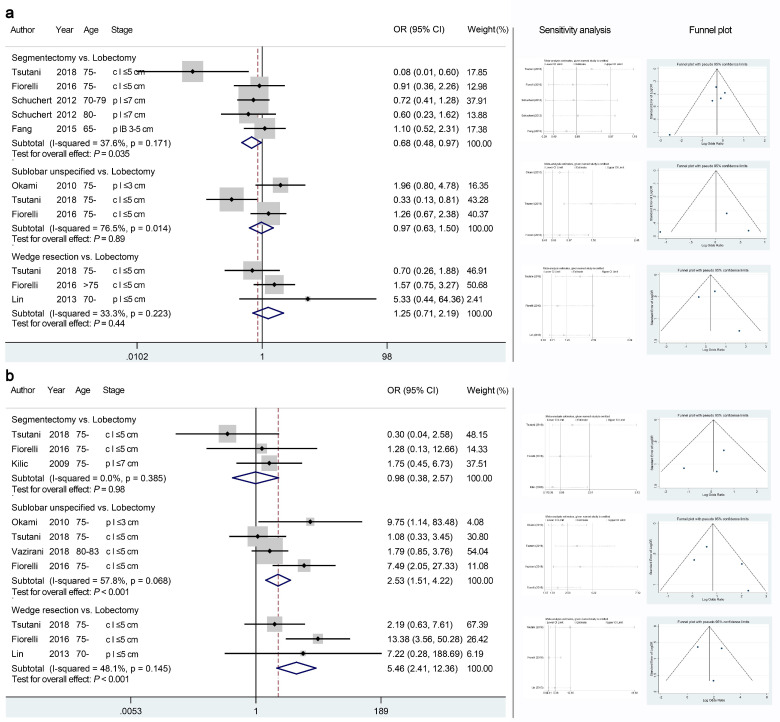
Meta-analyses comparing the overall recurrence risk (**a**) and local recurrence risk (**b**) of sublobar resections with those of lobectomy. The right panel presents the outcomes of sensitivity analyses and the funnel plots corresponding to the forest plots shown in the left panel. OR, odds ratio; CI, confidence interval.

A total of 11 cohort studies reported the recurrence patterns of sublobar resections compared to lobectomy (Table 1). The quality of these recurrence-related quantitative analyses was mostly moderate (Table 2). The segmentectomy group showed a decreased risk of overall recurrence than the lobectomy group (Figure 4a, OR 0.68, 95% CI 0.48–0.97, *p* = 0.035; *I*^2^ = 37.6; Egger’s test, *p* = 0.16) while wedge resection showed no significant difference in this regard (OR 1.25, 95% CI 0.71–2.91, *p* = 0.44; *I*^2^ = 33.3; Egger’s test, *p* = 0.73). Regarding local recurrence (Figure 4b), the segmentectomy group showed a comparable risk to the lobectomy group (OR 0.98, 95% CI 0.38–2.57, *p* = 0.98; *I*^2^ = 0; Egger’s test, *p* = 0.53), while the wedge resection group showed a significantly increased risk compared to the lobectomy group (OR 5.46, 95% CI 2.41–12.36, *p* < 0.001; *I*^2^ = 48.1; Egger’s test, *p* = 0.89). In particular, the local recurrence rates gradually increased among the segmentectomy, unspecified sublobar resection, and wedge resection groups (vs. lobectomy group). Intriguingly, sublobar resections, especially wedge resection, showed a decreased risk of distant metastasis compared with lobectomy based on the pooled analyses (Supplemental Figure S2).

The direct comparison between segmentectomy and wedge resection demonstrated a decreased risk of overall recurrence (OR 0.39, 95% CI 0.16–0.95, *p* = 0.039) and local recurrence (OR 0.11, 95% CI 0.02–0.51, *p* = 0.004) but not distant metastasis in the segmentectomy group (Supplemental Figure S3).

### 3.5. Secondary Endpoints: Perioperative Morbidities

The meta-analyses (Supplemental Figure S4) demonstrated non-significantly shorter operative time (WMD-34.41 min, *p* = 0.075) and significantly less blood loss (WMD-102.68 mL, *p* < 0.001) in the sublobar resection groups than in the lobectomy group. The chest drainage day was also shorter in the sublobar resection groups (WMD-0.99 d, *p* = 0.075). Furthermore, both the segmentectomy and wedge resection groups showed shorter hospital stays than the lobectomy group (WMD-1.94 d, *p* < 0.001; WMD-2.60 d, *p* < 0.001, respectively).

Analyses of NCDB demonstrated a decreased 30-day mortality rate in the sublobar resection groups [5]. The meta-analysis of eight cohort studies also demonstrated a significantly decreased 30-day mortality rate in the sublobar resection groups (Supplemental Figure S5a, OR 0.49, *p* = 0.035). The pooled analysis of the SEER and NCDB databases confirmed a significantly decreased 90-day mortality rate in the sublobar resection groups (Supplemental Figure S5b, OR 0.83, *p* = 0.007). The meta-analyses (Supplemental Figure S5c,d) demonstrated decreased incidence rates of overall complications and severe complications in the sublobar resection groups than in the lobectomy group (OR 0.70, *p* = 0.020; OR 0.61, *p* = 0.041, respectively). Regarding the specific complications (Supplemental Figure S5e–i), the sublobar resection groups showed a significantly lower risk of pneumonia (OR 0.42, *p* = 0.025) and leak complications (OR 0.45, *p* = 0.016) with non-significantly lower risk of cardiac complications, atelectasis and empyema.

## 4. Discussion

This is the first systematic review and meta-analysis investigating the efficacies of segmentectomy and wedge resection in elderly patients with early-stage NSCLC. Segmentectomy showed comparable survival outcomes and recurrence patterns to lobectomy and was superior to wedge resection.

Previous database studies and meta-analyses supported good efficacies of segmentectomy for stage IA1-2 NSCLC and wedge resection for stage IA1 NSCLC [7,8,35]. The early disclosure of the Japanese trial JCOG0802/WJOG4607L at the 101st AATS Annual Meeting revealed better OS of segmentectomy than lobectomy for peripheral NSCLC with a clinical stage IA ≤ 2 cm and C/T ratio > 0.5, especially for those with an age ≥70 years; however, segmentectomy is associated with an increased risk of local recurrence. Generally, the results of our meta-analysis on elderly patients are almost equal to analyses of cohorts without a focus on elderly patients [7,8]. The expanded indication of segmentectomy for elderly patients with stage I NSCLC was revealed for the first time. In particular, segmentectomy and wedge resection achieved a CSS benefit for stage IA1 patients over 75 years compared to lobectomy. The comparable local recurrence rate between segmentectomy and lobectomy indicates enough parenchymal margin achieved by segmentectomy, which is much better than wedge resection [16]. Apart from this advantage, anatomic segmentectomy allowed better lymph node staging than wedge resection. Dissection along the segmental bronchus during segmentectomy allows adjacent hilar lymph nodes, leading to increased yields of N1 lymph nodes [16]. In contrast, wedge resection is a non-anatomic resection, and systematic lymph node dissection or sampling was not implemented among the included studies (Table 1), contributing to elevated local recurrence rates. However, segmentectomy is still reported with inferior lymph node dissection and upstaging compared to lobectomy in the real world [36]. Radical segmentectomy, i.e., anatomical segmentectomy with hilar and mediastinal lymph node dissection, should thus be emphasized for elderly patients with stage I NSCLC.

During the aging process, increased comorbidities and impaired cardiopulmonary function not only increase perioperative morbidities but also compromise survival prognosis. As previously reported, lung cancer-related death in elderly stage I NSCLC patients was less than that caused by heart disease and chronic obstructive pulmonary disease [3]. Despite this fact, the oncological outcomes of lobectomy and sublobar resections were superior to that of stereotactic body radiation therapy for elderly patients with early-stage NSCLC [5,37]. Although this meta-analysis demonstrated a decreased risk of perioperative morbidities in sublobar resections compared to lobectomy, segmentectomy was always comparable with lobectomy in these regards [9,38,39]. Considering the compromised metabolic stress caused by muscle depletion during the aging process, segmentectomy with less issue damage than lobectomy could be a better choice for elderly patients [40,41]. Segmentectomy was also superior to lobectomy in preserving postoperative pulmonary function and promoting postoperative quality of life [6,39,42]. Nevertheless, complex segmentectomy with an elevated risk of an air leak and other complications should be introduced cautiously for elderly patients [43].

The inequality in patients and disease characteristics between groups (Table 1) should be cautiously considered when interpreting the findings. Even among the elderly patients, the segmentectomy and wedge resection groups showed more advanced age than lobectomy [3,4]. Pulmonary function was reported to be inferior in sublobar groups, while the prevalence of chronic obstructive pulmonary disease increased in these groups. These factors detrimentally impact the prognosis after surgical resection. In contrast, the tumor diameter, as an important staging and prognostic factor, was generally greater in the lobectomy group than in the sublobar groups. This may account for the increased risk of distant metastasis in the lobectomy group than in the sublobar resection groups. These inequalities may derive from the fact that sublobar resections were usually performed for small peripheral tumors or cases with impaired cardiopulmonary function (Table 1). However, it is not scientific to speculate that these factors will neutralize when comparing the efficacies of sublobar resections and lobectomy. Multidimensional subgroup analysis should be a valuable way to solve these uncertainties, but it cannot be achieved without the original data. This study only demonstrated comparable efficacy between segmentectomy and lobectomy in elderly patients with overall stage I NSCLC, while segmentectomy showed the potential to achieve better efficacy than lobectomy for elderly patients with earlier cancer stages. Based on these clinical warrants, we have started a multicenter randomized controlled trial comparing the efficacy of segmentectomy and wedge resection with lobectomy in stage IA patients over 70 years (NCT02360761) [17]. The study is ongoing and is anticipated to provide more evidence to answer these questions.

Other limitations should also be highlighted. The studies included were mostly retrospectively conducted, although several studies were derived from prospective databases. The verified definitions of elderly patients and the varied versions of the TNM system among the included studies compromised the stability and feasibility of the findings. While confounding factors were worrisome, we did need to rely on retrospective studies until the first randomized controlled trial on the subject became available.

## 5. Conclusions

This study revealed good application prospects of segmentectomy for elderly patients with stage I NSCLC. Segmentectomy showed comparable survival outcomes and recurrence patterns with lobectomy for this population, while wedge resection showed inferior outcomes. Anatomic segmentectomy with radical lymphadenectomy represents an alternative for elderly patients with early-stage NSCLC. The inherent limitations should be considered when interpreting these findings. The ongoing clinical trial will provide more evidence answering the question of segmentectomy versus lobectomy in elderly patients with early-stage NSCLC.

## Figures and Tables

**Table 1:** Characteristics of included studies.

Author Year	Database/Country	Study Design	Study Period	Female (%)	Age Cutoff	Cancer Stage (TNM)	Surgical Resections				Systematic Lymph Node Dissection/Sampling	Reasons for Sub	Patient Characteristics ^†^					Disease Characteristics ^†^				Extracted Endpoints
							Lob	Sub	Seg	Wed			Age	FEV1	DLCO	COPD	OCM	Size	*p* Stage	Histology	LNR	
***Database studies***																						
Wang et al. 2020	SEER	Retro	1998–2016	55.1	70-	c I ≤ 3 cm (TNM v.8)	3279	2918	620	2298	NR	NR	Y					Y	Y	N		OS, CSS
Onaitis et al. 2018	STS-GTSD	Retro	2002–2013	NR	65-	c I (NR)	A total of 20,635				NR	NR										OS
Stokes et al. 2018	NCDB	Retro	2004–2013	55.1	75-	c I ≤ 5 cm (TNM v.7)	11,993	4537	NR	NR	NR	NR										Mortality
Shirvani et al. 2015	SEER	Retro	2003–2009	53.4	66-	*p* I ≤ 5 cm (TNM v.7)	7215	1496	NR	NR	NR	NR	Y			Y	Y	Y	Y	N	Y	Mortality
***Cohort studies***																						
Mimae et al. 2020	Japan	Retro PM	2010–2016	46.6	80-	c I ≤ 2 cm (TNM v.8)	21	37	9	28	Lob/Seg: Yes Wed: No	I, C	N	N			N	N	N	N	Y	OS, DFS
Chen et al. 2018 ^‡^	China	Retro	2009–2015	56.5	65-	*p* I ≤ 2 cm (TNM v.8)	442	224	58	166	Lob/Seg: Yes Wed: No	I, C	Y					N		N		OS, DFS
Tsutani et al. 2018	Japan	Retro PM	2007–2015	42.4	75-	c I ≤ 5 cm (TNM v.7)	106	99	56	43	NR	I, C	N	N	Y		N	Y	Y	N		OS, DFS, recurrence, complications
Vazirani et al. 2018	Australia	Retro	2005–2016	NR	80-	c I ≤ 5 cm (TNM v.7)	121	79	34	45	Lob/Seg: Yes Wed: No	NR		N	N							OS, recurrence, mortality
Qiu et al. 2017	China	Retro	2006–2012	29.8	65-	*p* I ≤ 5 cm (TNM v.7)	206	39	NR	NR	Lob/Seg: Yes Wed: No	I, C	N	Y		Y	N	Y	Y	Y		OS, DFS, mortality, complications
Fiorelli et al. 2016	Italy	Retro PM	2006–2012	38.5	75-	c I ≤ 5 cm (TNM v.7)	149	90	39	51	Lob/Seg: Yes Wed: No	C	N	Y	Y	Y	Y	N	Y	N	Y	OS, CSS, DFS, recurrence, mortality, complications
Dell’Amore et al. 2015	Italy	Retro	2000–2010	20.5	80-	c I ≤ 3 cm (TNM v.7)	29	27			NR	NR	Y	Y	Y	N	N			N		OS, mortality, complications
Fang et al. 2015	China	Retro	2008–2010	52.1	65-	*p* IB 3–5 cm (TNM v.7)	126	-	116	-	Lob/Seg: Yes	C						N	N	N	N	OS, DFS, recurrence, complications
Liu et al. 2014	China	Retro	2004–2010	42.5	70-	*p* I ≤ 5 cm (TNM v.7)	122	45	NR	NR	NR	C										OS
Lin et al. 2013 ^‡^	China	Retro	2008–2012	38.3	70-	*p* I ≤ 5 cm (TNM v.7)	33	-	-	14	Lob/Seg: Yes Wed: No	C	N					N	N	N		Recurrence, mortality, complications
Warwick et al. 2013	UK	Retro PM	2001–2011	50.5	70-	*p* I ≤ 5 cm (TNM v.7)	152	-	-	83	Lob: Yes Wed: No	C	Y	Y		Y	Y	N	N			OS, mortality
Okada et al. 2012	Japan	Retro	1996–2008	31.8	80-	c I ≤ 7 cm (TNM v.6)	14	20	7	13	Lob/Seg: Yes Wed: No	C										OS, CSS
Schuchert et al. 2012 ^‡^	USA	Retro	1999–2010	51.4	70-	*p* I ≤ 7 cm (TNM v.6)	290	-	171	-	Lob/Seg: Yes	I, C	N	Y	Y		N	Y		N	Y	Recurrence, mortality, complications
Okami et al. 2010	Japan	Retro	1991–2007	35.8	75-	*p* I ≤ 3 cm (TNM v.7)	82	54	33	21	Lob/Seg: Yes Wed: No	C	N					N		Y		OS, recurrence, complications
Kilic et al. 2009 ^‡^	USA	Retro	2002–2007	50.0	75-	*p* I ≤ 7 cm (TNM v.6)	106	-	78	-	Lob/Seg: Yes	I, C	N	N	N	Y	Y	Y		N	Y	OS, DFS, recurrence, mortality, complications
Ghosh et al. 2003	UK	Retro	1991–2001	42.3	70-	*p* T1N0 (TNM v.6)	149	-	-	47	Lob: Yes Wed: No	C	N	Y						N		OS, Mortality, complications

^†^ Significant difference between lobectomy and sublobar resection groups present (Y) or not present (N), while the blank area indicated data not reported. ^‡^ Although the study by Chen et al. partially overlapped with that by Lin et al., different endpoints were extracted and analyzed, as did the studies by Schuchert et al. and Kilic et al. C, compromised; COPD, chronic obstructive pulmonary disease; CSS, cancer-specific survival; DFS, disease-free survival; DLCO, carbon monoxide diffusing capacity; FEV1, forced expiratory volume in the first second; I, intentional; LNR, lymph node resection; Lob, lobectomy; NCDB, National Cancer Database; NR, not reported; OCM, other comorbidities; OS, overall survival; PM, propensity score-matched; SEER, Surveillance, Epidemiology, and End Results database; Seg, segmentectomy; STS-GTSD, STS General Thoracic Surgery Database; Sub, sublobar resection; TNM, tumor, node, metastasis staging system; Wed, wedge resection.

**Table 2:** GRADE evidence profile: meta-analyses of surgical resections and endpoints.

Outcomes	Comparison	No. of Studies	Certainty Assessment					Effect	Quality	Forest Plot
			limitations	Inconsistency	Indirectness	Imprecision	Publication Bias	HR/OR (95% CI)		
OS 1 ^†^	Seg vs. Lob	3 [3,11]	Serious	Not serious	Not serious	Not serious	Undetected	1.07 (0.98–1.18)	⊕⊕⊕○ (Moderate)	Figure 2a
	Wed vs. Lob	3 [3,11]	Serious	Not serious	Not serious	Not serious	Undetected	1.28 (1.22–1.35)	⊕⊕⊕○ (Moderate)	Figure 2a
OS 2 ^†^	Seg vs. Lob	5 [12,24,25,30,33]	Serious	Not serious	Not serious	Not serious	Undetected	1.00 (0.78–1.27)	⊕⊕⊕○ (Moderate)	Figure 2b
	Sub vs. Lob	10 [12,13,14,22,23,24,26,27,30,32]	Serious	Not serious	Not serious	Not serious	Undetected	1.18 (0.97–1.43)	⊕⊕⊕○ (Moderate)	Figure 2b
	Wed vs. Lob	8 [12,22,23,24,29,30,34]	Serious	Not serious	Not serious	Not serious	Undetected	1.13 (0.91–1.40)	⊕⊕⊕○ (Moderate)	Figure 2b
CSS	Seg vs. Lob	8 [3,24,30]	Serious	Serious	Not serious	Not serious	Undetected	1.01 (0.88–1.17)	⊕⊕○ ○ (Low)	Figure 3a
	Sub vs. Lob	3 [3,24,30]	Serious	Serious	Not serious	Not serious	Undetected	1.02 (0.93–1.12)	⊕⊕○ ○ (Low)	Figure 3a
	Wed vs. Lob	8 [3,24,30]	Serious	Serious	Not serious	Not serious	Undetected	1.17 (1.06–1.30)	⊕⊕○ ○ (Low)	Figure 3a
DFS	Seg vs. Lob	4 [12,24,25,33]	Serious	Not serious	Not serious	Not serious	Undetected	1.04 (0.80–1.34)	⊕⊕⊕○ (Moderate)	Figure 3b
	Sub vs. Lob	5 [12,13,14,23,24]	Serious	Not serious	Not serious	Not serious	Undetected	1.07 (0.85–1.35)	⊕⊕⊕○ (Moderate)	Figure 3b
	Wed vs. Lob	4 [12,13,24]	Serious	Not serious	Not serious	Not serious	Undetected	1.44 (1.01–2.05)	⊕⊕⊕○ (Moderate)	Figure 3b
Overall recurrence	Seg vs. Lob	5 [14,24,25,31]	Serious	Not serious	Not serious	Not serious	Undetected	0.68 (0.48–0.97)	⊕⊕⊕○ (Moderate)	Figure 4a
	Sub vs. Lob	3 [14,24,32]	Serious	Serious	Not serious	Not serious	Undetected	0.97 (0.63–1.50)	⊕⊕○ ○ (Low)	Figure 4a
	Wed vs. Lob	3 [14,24,28]	Serious	Not serious	Not serious	Not seri2295ous	Undetected	1.25 (0.71–2.19)	⊕⊕⊕○ (Moderate)	Figure 4a
Local recurrence	Seg vs. Lob	3 [14,24,33]	Serious	Not serious	Not serious	Not serious	Undetected	0.98 (0.38–2.57)	⊕⊕⊕○ (Moderate)	Figure 4b
	Sub vs. Lob	4 [14,22,24,32]	Serious	Not serious	Not serious	Not serious	Undetected	2.53 (1.51–4.22)	⊕⊕⊕○ (Moderate)	Figure 4b
	Wed vs. Lob	3 [14,24,28]	Serious	Not serious	Not serious	Not serious	Undetected	5.46 (2.41–12.4)	⊕⊕⊕○ (Moderate)	Figure 4b
Distant metastasis	Seg vs. Lob	3 [14,24,33]	Serious	Not serious	Not serious	Not serious	Undetected	0.49 (0.26–0.91)	⊕⊕⊕○ (Moderate)	Sup-Figure S2
	Sub vs. Lob	3 [14,24,32]	Serious	Serious	Not serious	Not serious	Undetected	0.46 (0.26–0.81)	⊕⊕○ ○ (Low)	Sup-Figure S2
	Wed vs. Lob	3 [14,24,28]	Serious	Not serious	Not serious	Not serious	Undetected	0.30 (0.12–0.78)	⊕⊕⊕○ (Moderate)	Sup-Figure S2

^†^ Meta-analysis of overall survival using database studies (1) or cohort studies (2). GRADE, Grading of Recommendation Assessment, Development, and Evaluation (GRADE) system. CI, confidence interval; CSS, cancer-specific survival; DFS, disease-free survival; HR, hazard rate; N, negative; OR, odds ratio; OS, overall survival; Seg, segmentectomy; Sub, sublobar resection; Wed, wedge resection.

## Data Availability

All data generated or analyzed during this study are included in this published article and its supplementary information files.

## Supplementary Materials

The following supporting information can be downloaded at: https://www.mdpi.com/article/10.3390/jcm11020294/s1. Figure S1: Meta-analyses comparing the survival outcomes of segmentectomy with wedge resection. HR, hazard ratio; Cl, confidence interval. Figure S2: Meta-analyses comparing the distant metastasis risk of sublobar resections with lobectomy. OR, odds ratio; Cl, confidence interval. Figure S3: Meta-analyses comparing the recurrence patterns of segmentectomy with wedge resection. OR, odds ratio; Cl, confidence interval. Figure S4: Meta-analyses comparing the operative time (a), blood loss (b), chest drainage day (c), and hospital stay (d) of sublobar resections groups with those of lobectomy group. OR, odds ratio; Cl, confidence interval. Figure S5: Meta-analyses comparing the risk of perioperative morbidities between sublobar resection groups and lobectomy group. (a) 30-day mortality; (b) 90-day mortality; (c) overall complications; (d) severe complications; (e) pneumonia; (f) leak complications; (g) cardiac complications; (h) atelectasis; (i) empyema. OR, odds ratio; Cl, confidence interval. Table S1: The search strategy for the PubMed database (title and abstract) using search terms. Table S2: List of excluded studies with reasons for exclusion. Table S3: Quality assessment of enrolled studies according to the Newcastle-Ottawa Scale (NOS) for cohort studies.
