# Supplementary material for: Segmentectomy and Wedge Resection for Elderly Patients with Stage I Non-Small Cell Lung Cancer: A Systematic Review and Meta-Analysis
Source: J Clin Med. 2022 Jan 6;11(2):294. doi: 10.3390/jcm11020294 (PMC8782039; doi:10.3390/jcm11020294)
Supplement: Supplementary file 1 [file jcm-11-00294-s001.zip › jcm-1520030-supplementary.pdf]

Supplemental Tables and Figures

Supplemental Table S1: Page. 2
Supplemental Table S2: Page. 3-5
Supplemental Table S3: Page. 6
Supplemental Figure S1: Page. 7
Supplemental Figure S2: Page. 8
Supplemental Figure S3: Page. 9
Supplemental Figure S4: Page. 10
Supplemental Figure S5: Page. 11

Supplemental Table S1 The search strategy for the PubMed database (title and abstract) using search terms.

Set #	Search for	Results (April 1st 2021)
#1	((lung[Title/Abstract]) OR (respiratory[Title/Abstract])) OR (pulmonary[Title/Abstract])	1,428,054
#2	((((((cancer[Title/Abstract]) OR (carcinoma[Title/Abstract])) OR (adenocarcinoma[Title/Abstract])) OR (neoplasm[Title/Abstract])) OR (malignancy[Title/Abstract])) OR (ground-glass opacity[Title/Abstract])) OR (nodule[Title/Abstract])	2,438,579
#3	lobectomy[Title/Abstract]	19,674
#4	(((segmentectomy[Title/Abstract]) OR (wedge resection[Title/Abstract])) OR (sublobar resection[Title/Abstract])) OR (sublobectomy[Title/Abstract])	7,441
#5	(((age[Title/Abstract]) OR (old[Title/Abstract])) OR (elderly[Title/Abstract])) OR (octogenarian[Title/Abstract])	3,467,701
#6	((((survival[Title/Abstract]) OR (mortality[Title/Abstract])) OR (morbidity[Title/Abstract])) OR (complication[Title/Abstract])) OR (recurrence[Title/Abstract])	2,306,384
#7	(((((#1 AND #2) AND #3) AND #4) AND #5) AND #6)	496

Supplemental Table S2. List of excluded studies with reasons for exclusion

First author, year	Reasons	Reference
Heiden BT, 2021	No independent data of elderly patients	Heiden BT, Eaton DB Jr, Engelhardt KE, Chang SH, Yan Y, Patel MR, Kreisel D, Nava RG, Meyers BF, Kozower BD, Puri V. Analysis of Delayed Surgical Treatment and Oncologic Outcomes in Clinical Stage I Non-Small Cell Lung Cancer. JAMA Netw Open. 2021 May 3;4(5):e2111613. doi: 10.1001/jamanetworkopen.2021.11613. PMID: 34042991; PMCID: PMC8160592.
Zhang B, 2021	Overlapping	Zhang B, Liu R, Ren D, Li X, Wang Y, Huo H, Zhu S, Chen J, Song Z, Xu S. Comparison of Lobectomy and Sublobar Resection for Stage IA Elderly NSCLC Patients (≥70 Years): A Population-Based Propensity Score Matching's Study. Front Oncol. 2021 May 7;11:610638. doi: 10.3389/fonc.2021.610638. PMID: 34026604; PMCID: PMC8139614.
Gooseman MR, 2021	No independent data of elderly patients	Gooseman MR, Falcoz PE, Decaluwe H, Szanto Z, Brunelli A; ESTS Database Committee. Morbidity and mortality of lung resection candidates defined by the American College of Chest Physicians as 'moderate risk': an analysis from the European Society of Thoracic Surgeons database. Eur J Cardiothorac Surg. 2021 Mar 24:ezab028. doi: 10.1093/ejcts/ezab028. Epub ahead of print. PMID: 33760020.
Lou X, 2021	No comparisons between lobectomy and sublobar resection	Lou X, Sanders A, Wagh K, Binongo JN, Sancheti M, Javidfar J, Pickens A, Fernandez F, Force S, Khullar O. Safety and Feasibility of Thoracoscopic Lung Resection for Non-Small-Cell Lung Cancer in Octogenarians. Innovations (Phila). 2021 Jan-Feb;16(1):68-74. doi: 10.1177/1556984520971620. Epub 2020 Nov 27. PMID: 33245249.
de Ruiter JC, 2020	No comparisons between lobectomy and sublobar resection	de Ruiter JC, Heineman DJ, Daniels JM, van Diessen JN, Damhuis RA, Hartemink KJ. The role of surgery for stage I non-small cell lung cancer in octogenarians in the era of stereotactic body radiotherapy in the Netherlands. Lung Cancer. 2020 Jun;144:64-70. doi: 10.1016/j.lungcan.2020.04.005. Epub 2020 Apr 19. PMID: 32371262.
Yi E, 2020	No independent data of elderly patients	Yi E, Cho S, Kim K, Jheon S. Comparison of survival outcomes between sublobar resection and lobectomies in early-stage lung adenocarcinoma by propensity score matching analysis. Indian J Thorac Cardiovasc Surg. 2020 Jul;36(4):382-387. doi: 10.1007/s12055-019-00897-w. Epub 2020 Mar 3. PMID: 33061146; PMCID: PMC7525899.
Fan X, 2020	No independent data of elderly patients	Fan X, Liang Y, Bai Y, Yang C, Xu S. Conditional survival rate estimates of lobectomy, segmentectomy and wedge resection for stage IA1 non-small cell lung cancer: A population-based study. Oncol Lett. 2020 Aug;20(2):1607-1618. doi: 10.3892/ol.2020.11713. Epub 2020 Jun 9. PMID: 32724402; PMCID: PMC7377117.
Yaldız D, 2019	No independent data of elderly patients	Yaldız D, Yakut FC, Örs Kaya Ş, Gürsoy S, Yaldız MS. The Role of Sublobar Resection in T1 N0 Non-Small-Cell Pulmonary Carcinoma. Turk Thorac J. 2020 Sep;21(5):308-313. doi: 10.5152/TurkThoracJ.2019.19064. Epub 2020 Sep 1. PMID: 33031721; PMCID: PMC7544409.
Stiles BM, 2019	Overlapping	Stiles BM, Mao J, Harrison S, Lee B, Port JL, Altorki NK, Sedrakyan A. Sublobar resection for node-negative lung cancer 2-5 cm in size. Eur J Cardiothorac Surg. 2019 Nov 1;56(5):858-866. doi: 10.1093/ejcts/ezz146. PMID: 31168591.
Harrison S, 2018	Review	Harrison S, Stiles B, Altorki N. What is the role of wedge resection for T1a lung cancer? J Thorac Dis. 2018 Apr;10(Suppl 10):S1157-S1162. doi: 10.21037/jtd.2018.03.188. PMID: 29785289; PMCID: PMC5949395.
Hattori A, 2017	No independent data of elderly patients	Hattori A, Matsunaga T, Takamochi K, Oh S, Suzuki K. Surgical resection for clinical-Stage I radiological pure-solid lung cancer that met the current high risk criteria. Jpn J Clin Oncol. 2017 Jul 1;47(7):630-638. doi: 10.1093/jjco/hyx054. PMID: 28419333.
Smith CB, 2017	No comparisons between lobectomy and sublobar resection	Smith CB, Wolf A, Mhango G, Wisnivesky JP. Impact of Surgeon Volume on Outcomes of Older Stage I Lung Cancer Patients Treated via Video-assisted Thoracoscopic Surgery. Semin Thorac Cardiovasc Surg. 2017 Summer;29(2):223-230. doi: 10.1053/j.semtcvs.2017.01.013. Epub 2017 Feb 24. PMID: 28823334.

Razi SS, 2016	Overlapping	Razi SS, John MM, Sainathan S, Stavropoulos C. Sublobar resection is equivalent to lobectomy for T1a non-small cell lung cancer in the elderly: a Surveillance, Epidemiology, and End Results database analysis. J Surg Res. 2016 Feb;200(2):683-9. doi: 10.1016/j.jss.2015.08.045. Epub 2015 Sep 3. PMID: 26490227.
Iwata H, 2016	Review	Iwata H. Therapeutic strategy for small-sized lung cancer. Gen Thorac Cardiovasc Surg. 2016 Aug;64(8):450-6. doi: 10.1007/s11748-016-0676-6. Epub 2016 Jun 14. PMID: 27300350.
Gulack BC, 2016	No independent data of elderly patients	Gulack BC, Yang CJ, Speicher PJ, Yerokun BA, Tong BC, Onaitis MW, D'Amico TA, Harpole DH Jr, Hartwig MG, Berry MF. A Risk Score to Assist Selecting Lobectomy Versus Sublobar Resection for Early Stage Non-Small Cell Lung Cancer. Ann Thorac Surg. 2016 Dec;102(6):1814-1820. doi: 10.1016/j.athoracsur.2016.06.032. Epub 2016 Sep 1. PMID: 27592602; PMCID: PMC5140083.
Husain ZA, 2015	Overlapping	Husain ZA, Kim AW, Yu JB, Decker RH, Corso CD. Defining the High-Risk Population for Mortality After Resection of Early Stage NSCLC. Clin Lung Cancer. 2015 Nov;16(6):e183-7. doi: 10.1016/j.cllc.2015.04.007. Epub 2015 Apr 24. PMID: 25979646.
Ohtsuka T, 2015	No independent data of elderly patients	Ohtsuka T, Kamiyama I, Asakura K, Kohno M. Thirty-day outcomes after lobectomy or segmentectomy for lung cancer surgery. Asian Cardiovasc Thorac Ann. 2015 Sep;23(7):828-31. doi: 10.1177/0218492315589476. Epub 2015 Jun 12. PMID: 26071452.
Yano M, 2015	No independent data of elderly patients	Yano M, Yoshida J, Koike T, Kameyama K, Shimamoto A, Nishio W, Yoshimoto K, Utsumi T, Shiina T, Watanabe A, Yamato Y, Watanabe T, Takahashi Y, Sonobe M, Kuroda H, Oda M, Inoue M, Tanahashi M, Adachi H, Saito M, Hayashi M, Otsuka H, Mizobuchi T, Moriya Y, Takahashi M, Nishikawa S, Matsumura Y, Moriyama S, Nishiyama T, Fujii Y; Japanese Association for Chest Surgery. Survival of 1737 lobectomy-tolerable patients who underwent limited resection for cStage IA non-small-cell lung cancer. Eur J Cardiothorac Surg. 2015 Jan;47(1):135-42. doi: 10.1093/ejcts/ezu138. Epub 2014 Apr 3. PMID: 24699203.
Warwick R, 2013	No independent data of elderly patients	Warwick R, Mediratta N, Shackcloth M, Page R, McShane J, Shaw M, Poullis M. Wedge resection verses lobectomy for stage 1 non-small-cell lung cancer. Asian Cardiovasc Thorac Ann. 2013 Oct;21(5):566-73. doi: 10.1177/0218492312466861. Epub 2013 Jul 11. PMID: 24570559.
Fan J, 2013	No comparisons between lobectomy and sublobar resection	Fan J, Chang Z, Ye C, Zhao B, Shen G, Chai Y. Video-assisted thoracoscopic superior segmentectomy of the right lower lobe. J Thorac Dis. 2013 Aug;5 Suppl 3(Suppl 3):S287-8. doi: 10.3978/j.issn.2072-1439.2013.07.18. PMID: 24040541; PMCID: PMC3771586.
Dell'Amore A, 2013	No independent data of elderly patients with Stage I lung cancer	Dell'Amore A, Monteverde M, Martucci N, Sanna S, Caroli G, Stella F, Dell'Amore D, Rocco G. Early and long-term results of pulmonary resection for non-small-cell lung cancer in patients over 75 years of age: a multi-institutional study. Interact Cardiovasc Thorac Surg. 2013 Mar;16(3):250-6. doi: 10.1093/icvts/ivs473. Epub 2012 Nov 23. PMID: 23178392; PMCID: PMC3568799.
Zhang J, 2012	No independent data of elderly patients with Stage I lung cancer	Zhang J, Xue ZQ, Chu XY, Wang YX, Zhao JH, Xu C, Yin LG. Surgical treatment and prognosis of octogenarians with non-small cell lung cancer. Asian Pac J Trop Med. 2012 Jun;5(6):465-8. doi: 10.1016/S1995-7645(12)60079-0. PMID: 22575979.
Okada A, 2012	No comparisons between lobectomy and sublobar resection	Okada A, Hirono T, Watanabe T. Safety and prognosis of limited surgery for octogenarians with non-small-cell lung cancer. Gen Thorac Cardiovasc Surg. 2012 Feb;60(2):97-103. doi: 10.1007/s11748-011-0880-3. Epub 2012 Feb 12. PMID: 22327854.
Shirvani SM, 2012	overlapping	Shirvani SM, Jiang J, Chang JY, Welsh JW, Gomez DR, Swisher S, Buchholz TA, Smith BD. Comparative effectiveness of 5 treatment strategies for early-stage non-small cell lung cancer in the elderly. Int J Radiat Oncol Biol Phys. 2012 Dec 1;84(5):1060-70. doi: 10.1016/j.ijrobp.2012.07.2354. Epub 2012 Sep 11. PMID: 22975611; PMCID: PMC3776428.
Rivera C, 2011	No comparisons between lobectomy	Rivera C, Falcoz PE, Bernard A, Thomas PA, Dahan M. Surgical management and outcomes of elderly patients with early stage non-

	and sublobar resection	small cell lung cancer: a nested case-control study. Chest. 2011 Oct;140(4):874-880. doi: 10.1378/chest.10-2841. Epub 2011 Mar 24. PMID: 21436251.
Ilonen IK, 2011	No independent data of elderly patients	Ilonen IK, Räsänen JV, Knuuttila A, Salo JA, Sihvo EI. Anatomic thoracoscopic lung resection for non-small cell lung cancer in stage I is associated with less morbidity and shorter hospitalization than thoracotomy. Acta Oncol. 2011 Oct;50(7):1126-32. doi: 10.3109/0284186X.2011.555780. Epub 2011 Feb 11. PMID: 21314296.
Rivera C, 2011	Lack of endpoints	Rivera C, Dahan M, Bernard A, Falcoz PE, Thomas P. Surgical treatment of lung cancer in the octogenarians: results of a nationwide audit. Eur J Cardiothorac Surg. 2011 Jun;39(6):981-6. doi: 10.1016/j.ejcts.2010.09.022. Epub 2010 Oct 27. PMID: 21030267.
Boffa DJ, 2008	No independent data of elderly patients	Boffa DJ, Allen MS, Grab JD, Gaissert HA, Harpole DH, Wright CD. Data from The Society of Thoracic Surgeons General Thoracic Surgery database: the surgical management of primary lung tumors. J Thorac Cardiovasc Surg. 2008 Feb;135(2):247-54. doi: 10.1016/j.jtcvs.2007.07.060. Epub 2007 Dec 21. PMID: 18242243.
Mery CM, 2005	Overlapping	Mery CM, Pappas AN, Bueno R, Colson YL, Linden P, Sugarbaker DJ, Jaklitsch MT. Similar long-term survival of elderly patients with non-small cell lung cancer treated with lobectomy or wedge resection within the surveillance, epidemiology, and end results database. Chest. 2005 Jul;128(1):237-45. doi: 10.1378/chest.128.1.237. PMID: 16002941.
Pagni S, 1997	No comparisons between lobectomy and sublobar resection	Pagni S, Federico JA, Ponn RB. Pulmonary resection for lung cancer in octogenarians. Ann Thorac Surg. 1997 Mar;63(3):785-9. doi: 10.1016/s0003-4975(96)01150-2. PMID: 9066402.
Naunheim KS, 1994	No comparisons between lobectomy and sublobar resection	Naunheim KS, Kesler KA, D'Orazio SA, Fiore AC, Judd DR. Lung cancer surgery in the octogenarian. Eur J Cardiothorac Surg. 1994;8(9):453-6. doi: 10.1016/1010-7940(94)90013-2. PMID: 7811476.

Supplemental Table S3 Quality assessment of enrolled studies according to the Newcastle-Ottawa Scale (NOS) for cohort studies.

Study, year	Selection				Comparability	Outcome			Total score
	Representativeness of the exposed cohort	Selection of the non-exposed cohort	Ascertainment of exposure	Demonstration that outcome of interest was not present at start of study	Comparability of cohorts on the basis of the design or analysis	Assessment of outcome	Was follow-up long enough for outcomes to occur	Adequacy of follow up of cohorts	
**Database studies**									
Wang, 2020	★	★	★	☆	★★	★	★	☆	7
Onaitis, 2018	★	★	★	☆	☆★	★	★	☆	6
Stokes, 2018	★	★	★	☆	★★	★	★	★	8
Shirvani, 2015	★	★	★	☆	☆★	★	★	★	7
**Cohort studies**									
Mimae, 2020	★	★	★	☆	☆★	★	★	☆	6
Chen, 2018	★	★	★	☆	★★	★	★	☆	7
Tsutani, 2018	★	★	★	☆	★★	★	★	☆	7
Vazirani, 2018	★	★	★	☆	★☆	★	★	☆	6
Qiu, 2017	★	★	★	☆	★☆	★	★	★	7
Fiorelli, 2016	★	★	★	☆	★★	★	★	☆	7
Fang, 2015	★	★	★	☆	☆★	★	★	☆	6
Dell'Amore, 2015	★	★	★	☆	☆★	★	★	★	7
Liu, 2014	★	★	★	☆	★☆	★	★	☆	6
Lin, 2013	★	★	★	☆	★☆	★	★	☆	6
Warwick, 2013	★	★	★	☆	★★	★	★	★	8
Okada, 2012	★	★	★	☆	☆★	★	★	☆	6
Schuchert, 2012	★	★	★	☆	☆★	★	★	★	7
Okami, 2010	★	★	★	☆	☆★	★	★	☆	6
Kilic, 2009	★	★	★	☆	☆★	★	★	☆	6
Ghosh, 2003	★	★	★	☆	☆★	★	★	☆	6

A score of 7 or higher indicates a low risk of bias.

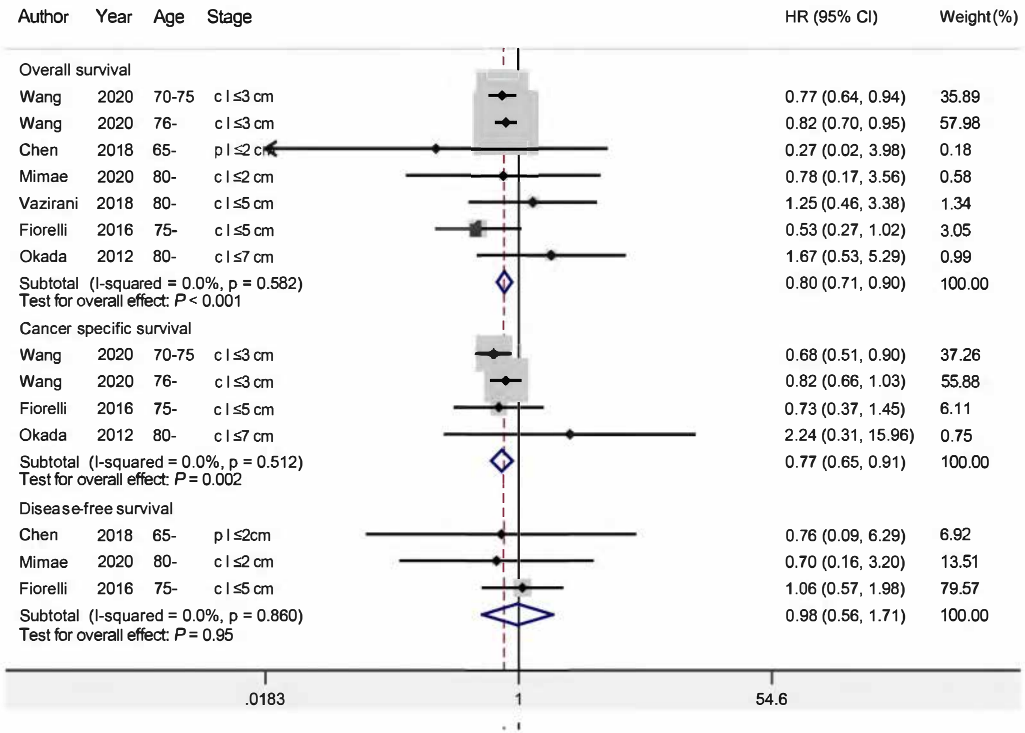

Supplemental Figure S1 Meta-analyses comparing the survival outcomes of segmentectomy with wedge resection. HR, hazard ratio; CI, confidence interval.

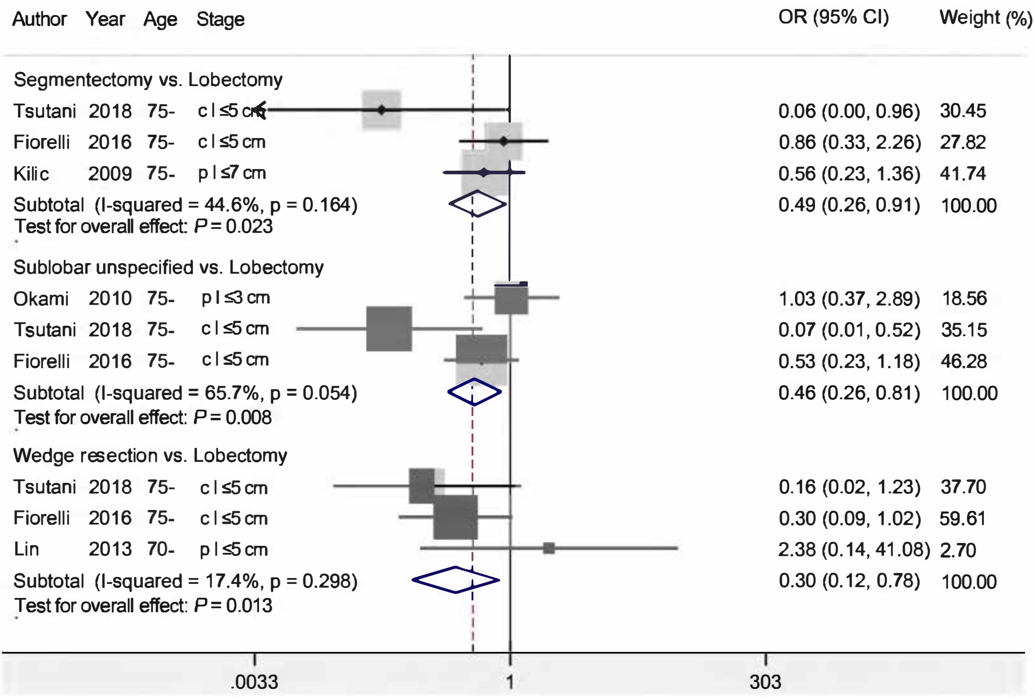

Supplemental Figure S2 Meta-analyses comparing the distant metastasis risk of sublobar resections with lobectomy. OR, odds ratio; CI, confidence interval.

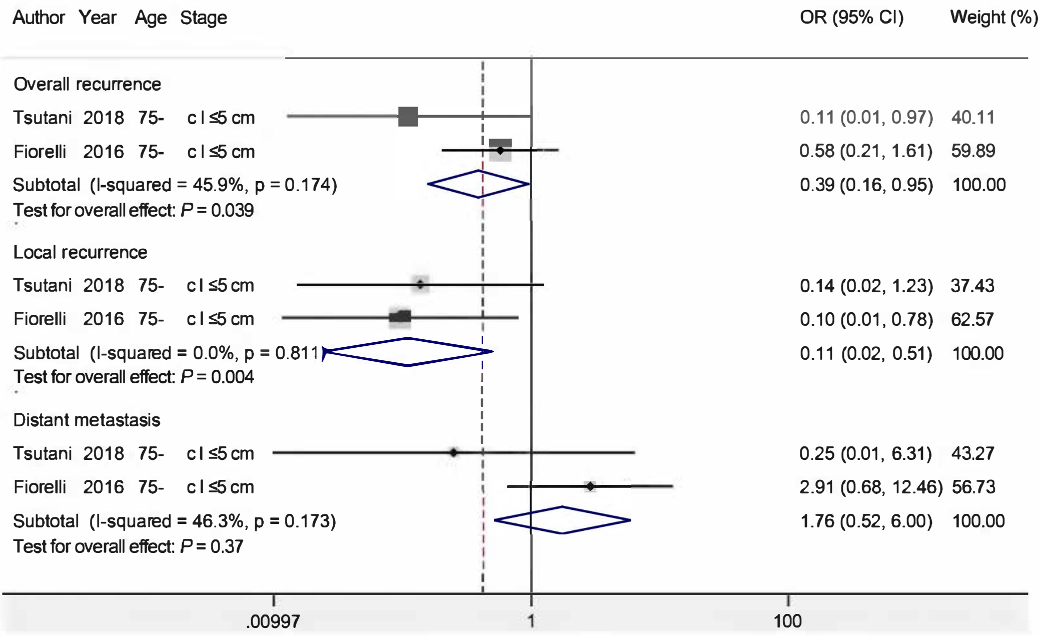

Supplemental Figure S3 Meta-analyses comparing the recurrence pattems of segmentectomy with wedge resection. OR, odds ratio; CI, confidence interval.

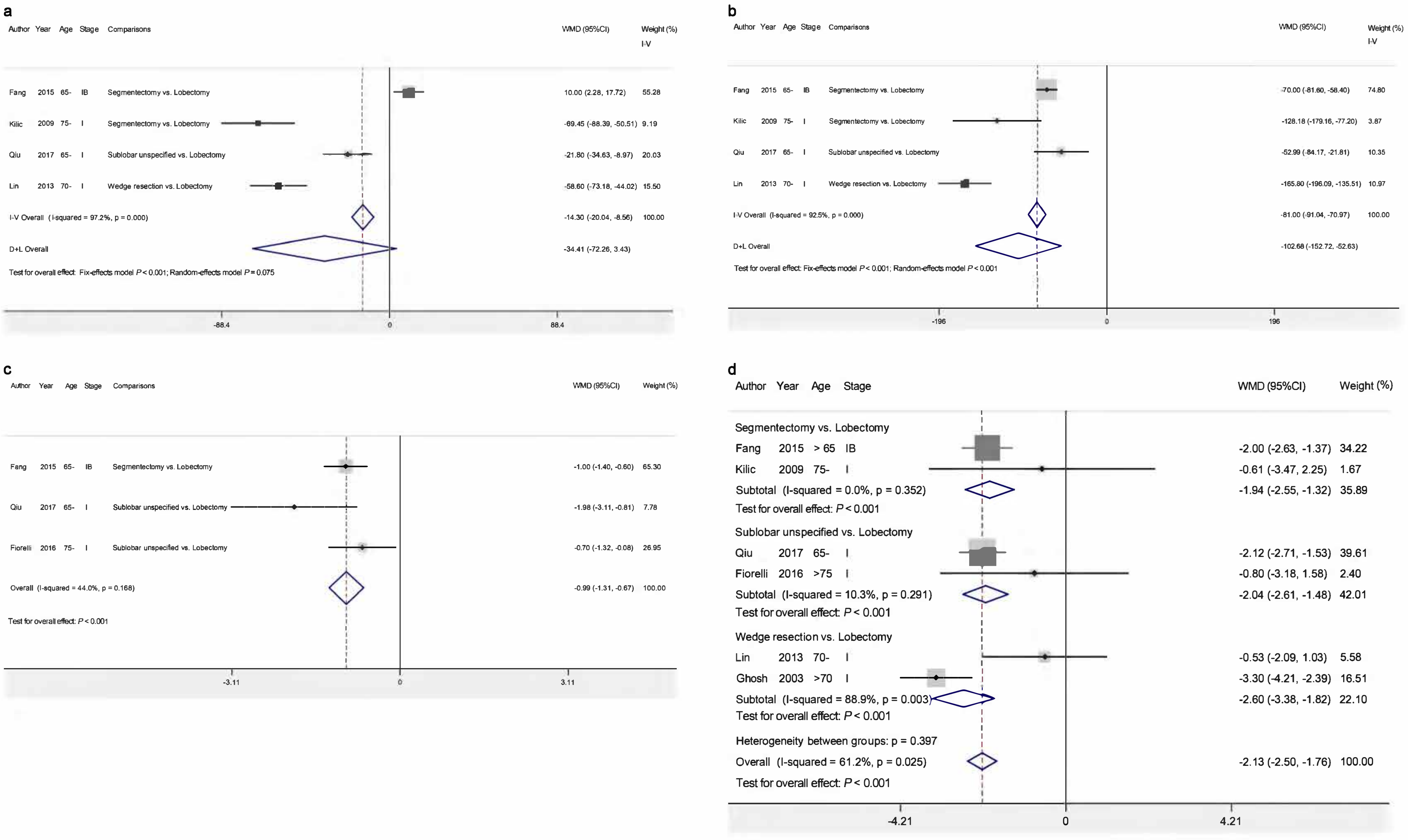

Supplemental Figure S4 Meta-analyses comparing the operative time (a), blood loss (b), chest drainage day (c), and hospital stay (d) of sublobar resections groups with those of lobectomy group. OR, odds ratio; CI, confidence interval.

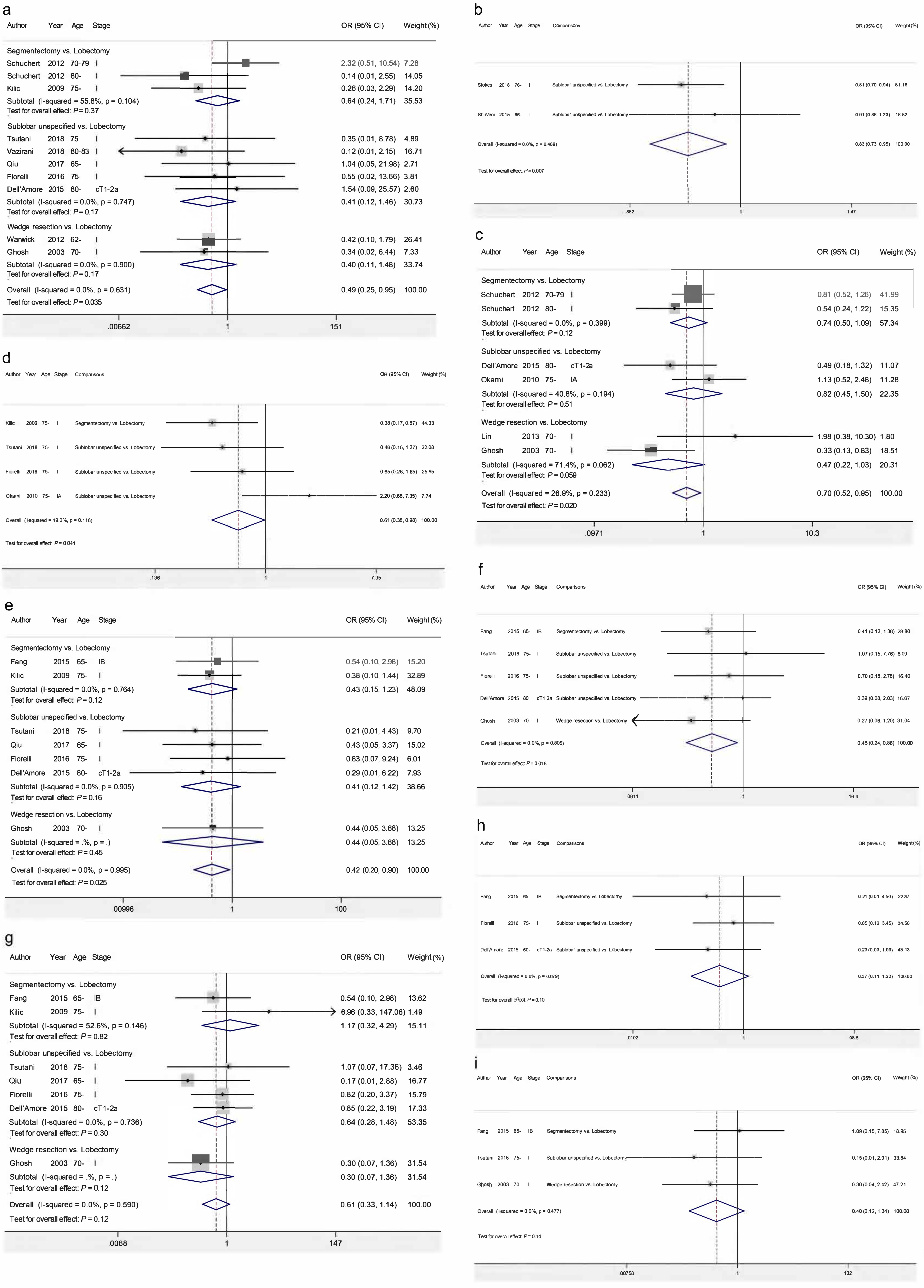

Supplemental Figure S5 Meta-analyses comparing the risk of perioperative morbidities between sublobar resection groups and lobectomy group. (a) 30-day mortality; (b) 90-day mortality; (c) overall complications; (d) severe complications; (e) pneumonia; (f) leak complications; (g) cardiac complications; (h) atelectasis; (i) empyema. OR, odds ratio; CI, confidence interval.
